# Presentation of Diagnostic Information to Doctors May Change Their Interpretation and Clinical Management: A Web-Based Randomised Controlled Trial

**DOI:** 10.1371/journal.pone.0128637

**Published:** 2015-07-06

**Authors:** Yoav Ben-Shlomo, Simon M. Collin, James Quekett, Jonathan A. C. Sterne, Penny Whiting

**Affiliations:** 1 School of Social & Community Medicine, University of Bristol, Canynge Hall, Bristol, United Kingdom; 2 Centre for Child & Adolescent Health, University of Bristol, Oakfield House, Oakfield Grove, Bristol, United Kingdom; 3 Doctors.net.uk, Milton Park, Abingdon, Oxfordshire, United Kingdom; 4 Kleijnen Systematic Reviews Ltd, York, United Kingdom; Instituto de Higiene e Medicina Tropical, PORTUGAL

## Abstract

**Background:**

There is little evidence on how best to present diagnostic information to doctors and whether this makes any difference to clinical management. We undertook a randomised controlled trial to see if different data presentations altered clinicians’ decision to further investigate or treat a patient with a fictitious disorder (“Green syndrome”) and their ability to determine post-test probability.

**Methods:**

We recruited doctors registered with the United Kingdom’s largest online network for medical doctors between 10 July and 6” November 2012. Participants were randomised to one of four arms: (a) text summary of sensitivity and specificity, (b) Fagan’s nomogram, (c) probability-modifying plot (PMP), (d) natural frequency tree (NFT). The main outcome measure was the decision whether to treat, not treat or undertake a brain biopsy on the hypothetical patient and the correct post-test probability. Secondary outcome measures included knowledge of diagnostic tests.

**Results:**

917 participants attempted the survey and complete data were available from 874 (95.3%). Doctors randomized to the PMP and NFT arms were more likely to treat the patient than those randomized to the text-only arm. (ORs 1.49, 95% CI 1.02, 2.16) and 1.43, 95% CI 0.98, 2.08 respectively). More patients randomized to the PMP (87/218–39.9%) and NFT (73/207–35.3%) arms than the nomogram (50/194–25.8%) or text only (30/255–11.8%) arms reported the correct post-test probability (p <0.001). Younger age, postgraduate training and higher self-rated confidence all predicted better knowledge performance. Doctors with better knowledge were more likely to view an optional learning tutorial (OR per correct answer 1.18, 95% CI 1.06, 1.31).

**Conclusions:**

Presenting diagnostic data using a probability-modifying plot or natural frequency tree influences the threshold for treatment and improves interpretation of tests results compared to text summary of sensitivity and specificity or Fagan’s nomogram.

## Introduction

Accurate diagnosis is fundamental to the provision of appropriate health care. The volume of diagnostic tests performed in the UK National Health Service (NHS) has increased dramatically in recent years due to technological innovation and an ageing population [1]. Doctors order diagnostic tests for a variety of reasons other than for making a diagnosis [2]. Research using cognitive psychology suggests that sensitivity and specificity are generally poorly understood by clinicians [3,4] and are often confused with predictive values [3,5,6]. Clinicians tend to overestimate the impact of a positive test result on the probability of disease [7,8], and this overestimation increases with decreasing pre-test probabilities of disease [8].

The most informative measures for clinicians may be estimates of the post-test probability of disease, which can be presented as a range corresponding to different pre-test probabilities. However, there has been little research into the most appropriate graphical, tabular and statistical summaries of test characteristics, and whether these have any influence on clinical management. Graphical displays (such as forest plots, (summary) receiver operator characteristics (S)ROC plots, likelihood ratio nomograms and probability modifying plots) are under-used in both primary diagnostic accuracy studies and systematic reviews [9]. Clinicians and medical students find interpretation of natural frequencies [10], the joint frequency of two events i.e. number of people with disease who have a positive test, easier than simple percentages or probabilities such as sensitivity or specificity [11]. Six previous trials [7,12–16] have compared health professionals’ ability to estimate post-test probability for different methods of presentation of diagnostic information. Four of these were small (<300 participants) and only one evaluated of a graphical method of presenting diagnostic information. In all but one of these studies the primary outcome was the ability to derive a post-test probability rather than the influence of the presentation on clinical care. The single study evaluating effects on clinical care [16] only provided data on sensitivity and specificity with and without definitions, and did not evaluate graphical presentations.

We conducted the first large randomised controlled trial (RCT) assessing the influence of different formats for presenting diagnostic test results on clinicians’ understanding and on reported clinical management.

## Methods

The study received ethical approval from the Frenchay Research Ethics Committee (10/H0107/8). Participants were recruited via a UK-based online network for medical doctors. Participants who completed the module were also given the option of completing a further tutorial (slide presentation, short video, or long video) on diagnostic tests, for which they could download a certificate of completion to add to their Continuing Professional Development (CPD) portfolio.

### Recruitment

Our study was advertised on the doctors.net.uk website from July to November 2012 using a banner headline. Doctors.net.uk is the UK’s largest online network for medical doctors, with a total membership of 198,000 of whom approximately 49,000 access the network during any one week. Dr Wendy Peek (general practitioner) also wrote a blog about the pros and cons of ordering diagnostic tests and included a link to the module. This blog is emailed to around 10,000 doctors. In addition, a banner advert was created which directed the individual doctors to a brief explanation of the study and a link to the module. Before proceeding with the web-based module, participants were asked to give their consent for their data from the study to be stored and used for research purposes by clicking a box on the web form. This was felt to be an appropriate method of obtaining consent by the ethics committee as there was no direct contact between the researchers and the participants. They were also given the option of providing contact details for entry into a prize draw. Participants were randomly allocated to one of four data presentation formats by means of an algorithm embedded in the web page source code using simple randomization. Participants were unaware (“blinded”) that they were randomly allocated to one of these formats.

### Nature of the intervention

**Fig 1:**
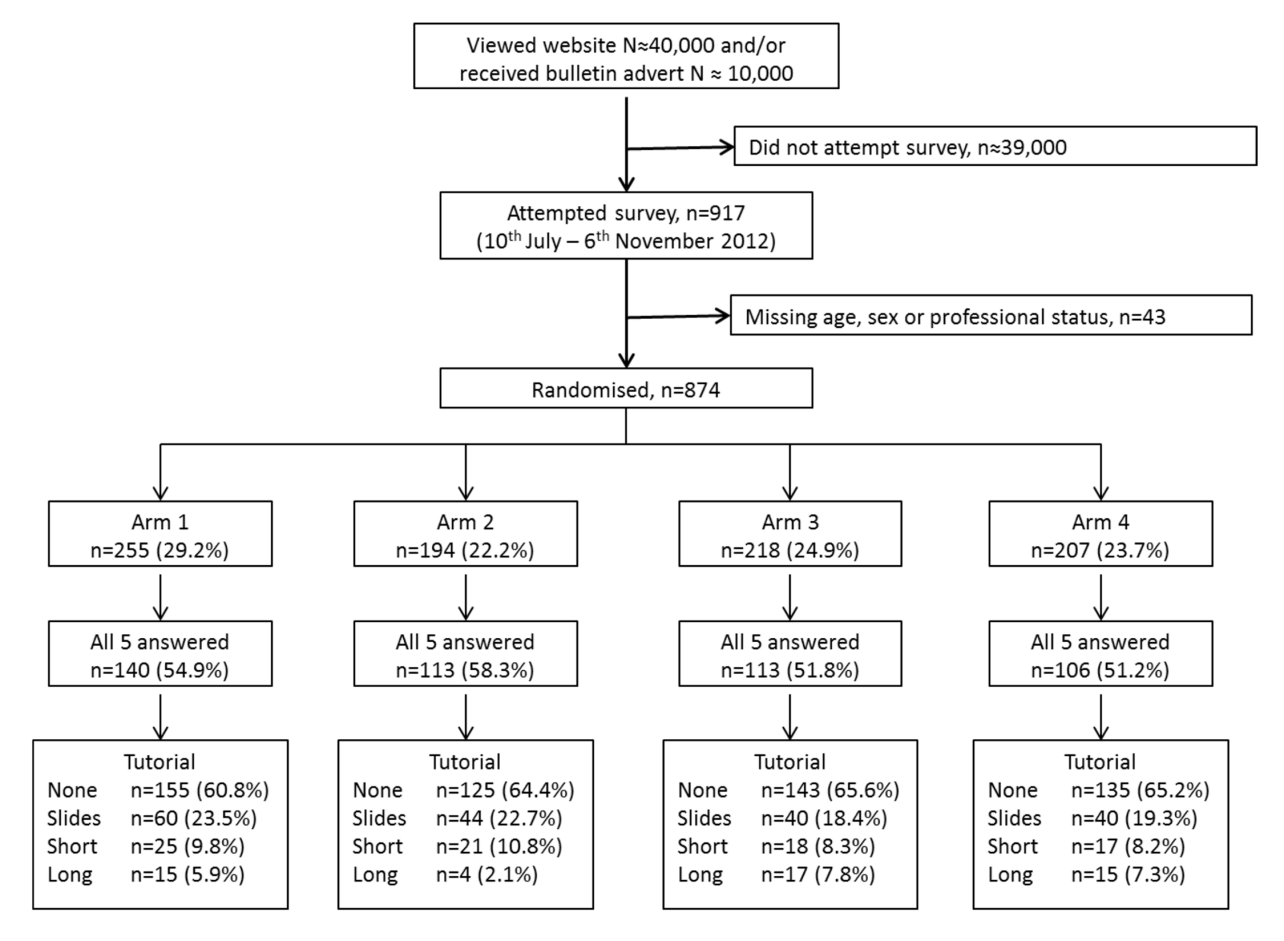
Different modes of data presentation to help interpret the results of the index test (A) Fagan’s nomogram (B) Probability modifying plot (C) Natural frequency tree.

We designed an interactive web-based module based on a case-vignette approach that has been used successfully elsewhere [7,17]. All participants were presented with a hypothetical scenario (**Table 1**) that we constructed. We chose this approach rather than using a real medical condition so that the participants could not be influenced by previous knowledge, experience or guidelines for any specific real clinical condition. Participants then proceeded to a web page which presented information about the accuracy of the “anti-celadon” test in one of four different formats:

- Short text summary as one might find in the abstract of a conventional journal article: “The anti-celadon test has a sensitivity of 60% and a specificity of 90%. It has a positive likelihood ratio of 6 and a negative likelihood ratio of 0.44 suggesting that it has greater potential to rule in Green syndrome rather than to rule out Green syndrome.”
- The same short text summary as for (a) supported by a nomogram (**Fig 1A**) for which we provided the following instructions “… enables you to convert the pre-test probability to a post-test probability by connecting the values for the pre-test probability and appropriate likelihood ratio and extrapolating a straight line to the post-test probability column.”
- Short text summary as for (a) (without comment on “greater potential to rule in”) supported by a probability-modifying plot (**Fig 1B**) with the following text—“allows you convert the pre-test probability to a post-test probability by drawing a vertical line from the appropriate pre-test probability on the x-axis and extrapolating a horizontal line to the y-axis (post-test probability) at the point where the line hits the appropriate positive or negative test result curve.”
- The same short text summary as for (a) but supported by a natural frequency tree [18,19] (**Fig 1C**) showing a “theoretical sample of 1000 patients who also have a pre-test probability of 50% similar to Debbie.”

**Table 1:** Hypothetical scenario and background information about “Green Syndrome.”

"Green syndrome" is a serious (hypothetical) chronic disease that presents with a period of mild illness, which, if left untreated, can progress to a more serious disease with a 5% risk of becoming a wheelchair user within 20 years. There is an effective treatment "Viridian" which can prevent progression of the syndrome. However, this is costly at £30,000 ($50,000 or €35,000) and has a high risk of side effects: 30% of patients suffer hair loss and 10% experience jaundice, although symptoms recover on cessation of treatment.
A 35 year old woman (Debbie) presents to you with new symptoms. Based on her clinical history, physical examination and symptoms, you really can't decide if she does or does not have "Green Syndrome". This means that your pre-test probability for disease is 50%.
A new test, the "anti-celadon test", which is cheap and based on a simple blood sample, has recently become available. Previously the only available test required a brain biopsy which had associated morbidity and very rarely mortality, but has 100% specificity and 95% sensitivity. You decide to order the anti-celadon test as you are unsure of the diagnosis. You find the key paper on the anti-celadon test to get further information on its accuracy.

### Primary outcome measures

All participants were asked the same two questions:

1. “The anti-celadon test for Debbie comes back positive. Given her pre-test probability of 50%, what is the new probability (0–100%) that Debbie has Green Syndrome?” (The correct answer to this question is 86%).
2. Would you decide to treat Debbie, not treat Debbie or order a brain biopsy?

Answers to question 1) could be entered into a text box. Three possible answers to question 2) were presented as mutually-exclusive tick-boxes: “Treat”; “Not treat”; and “Order brain biopsy”. Participants allocated to the natural frequency arm were also asked “How many of the patients who have a positive test result actually have Green Syndrome?” to see if they correctly used the data from the natural frequency tree.

### Other explanatory variables

We asked participants their professional status, specialty, age, sex, year of qualification, and country of practice. In addition they were asked to rank how confidently they rated their ability to interpret data on the performance of diagnostic tests (e.g. sensitivity and specificity) on a 5-point scale from 1 “not at all” to 5 “extremely confident” and whether they had received any postgraduate training in evidence-based medicine or clinical epidemiology.

### Baseline knowledge

After completing the case-based scenario, we evaluated participants’ baseline knowledge of diagnostic concepts by presenting them with five questions about a new diagnostic test with sensitivity 90%; specificity 50%; positive predictive value 33%; negative predictive value 95%. These were (1) What percentage of patients with the disease would be misdiagnosed by the test? (2) Given a positive test result, what is the probability of having the disease? (3) What is the false positive rate of this test? (4) What percentage of patients with a negative test will still have the disease? (5) In the main paper, the authors report that the positive likelihood ratio of this test is 1.8 and the negative likelihood ratio is 0.2. Is this test more useful for ruling out or ruling in the diagnosis of the disease?

Answers to questions 1 to 4 could be entered into a text box, which participants were instructed to leave blank for “don’t know”. Three possible answers to question 5) were presented as mutually-exclusive tick-boxes: “Ruling out”; “Ruling in”; and “Don’t know”. On the final web page, all participants were presented with answers to the 5 questions and their score. At this point participants could finish the module or could undertake further educational training by opting for an add-on tutorial.

### Optional tutorials

Participants were given the option of completing one of three further tutorials (developed by YBS and PW) on diagnostic test accuracy studies: a 40-minute lecture with audio and slides; a shorter (10 minute) lecture with audio and slides; or a slides-only version presenting the same material as in the 40-minute lecture but without the accompanying commentary. Each tutorial could be accessed by clicking on a hyperlink. The video tutorials are available on https://vimeo.com/44049265 (short video) and https://vimeo.com/41140560 (long tutorial). Participants were presented with the key learning points of the tutorial and were able to download a certificate of completion for their Continuing Professional Development (CPD) portfolio.

### Statistical analysis

We compared the distribution of categorical responses using chi-squared tests. We used logistic regression models to quantify associations (odds ratios, 95% confidence intervals and p-values) of trial arm with choice of management (treatment, no treatment or biopsy) and correct answer to the question on post-test probability as our main outcomes. We repeated analyses using a multivariable model to adjust for any imbalance in baseline covariates (gender, age group, postgraduate education, self-rated confidence and professional status). We also estimated effects of presentation on management after conditioning on the post-test probability modelled as a continuous variable (estimates were similar when conditioning for tertiles of post-test probability). Answers to questions were coded as correct (allowing 1% in either direction for rounding errors and because given the resolution of the probability-modifying plot, we felt most people who did this correctly would read 85% rather than 86%), incorrect, or blank/don’t know. We did a post-hoc sensitivity analysis allowing a greater margin of error (+/- 3%). A pre-study sample size calculation estimated that we would need a sample size of 800 participants (200 per arm) to detect differences of 11–15% in preference for treatment if the probability of treatment ranged between 20 to 80% with 80% power at 5% significance.

## Results

### Participants and randomisation

**Fig 2:**
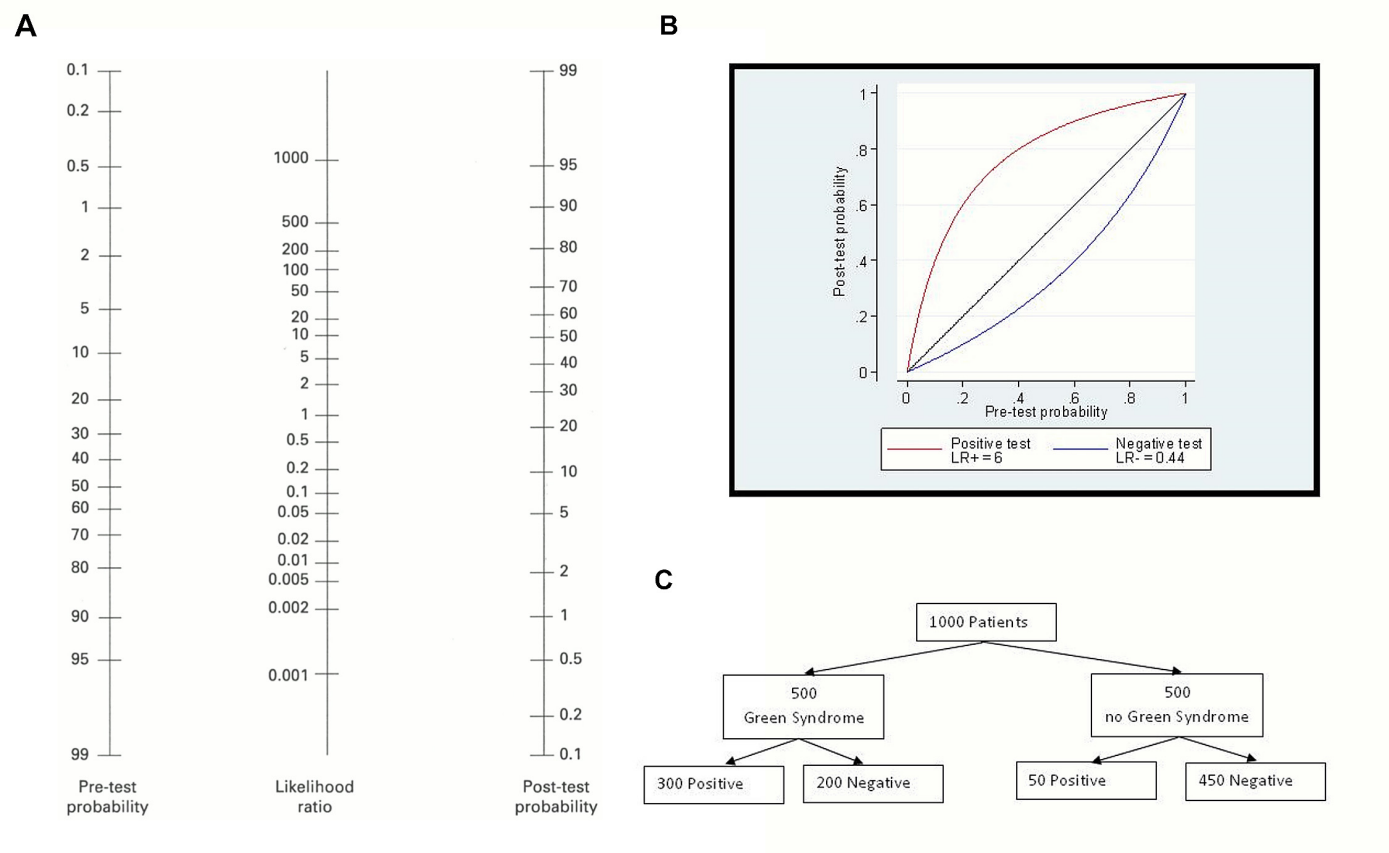
CONSORT flowchart.

We do not know precisely how many doctors viewed the banner advert, but we do know that 2,510 out of around 10,000 doctors opened the email from Dr Wendy Peek. The use of advertising banners is a standard in the website industry and can be used to reach the whole audience. Educational material on Doctors.net.uk is promoted by Dr Wendy Peek via a more targeted route of doctors who have previously expressed interest in educational materials and it was felt that this audience may be more receptive to participation in the study. In all, 917 doctors attempted the module between 10^th^ July 2012 and 6^th^ November 2012 and 874 doctors provided data on age, sex and professional status. Allocation to the four study arms is illustrated in **Fig 2**. There was a larger percentage of participants allocated to arm 1 (29.2%) compared to the average of the other three arms (23.4%).

Participant characteristics (sex, professional status, postgraduate training, and degree of confidence) were similar across the four arms (**Table 2**). The majority of participants were hospital consultants (31.7%) or trainee junior doctors (44.3%). There were more men (58.5%) than women, and just over half (55.1%) were under 40 years of age. All but 69 (7.9%) practiced in the UK (largest non-UK group from Norway), and 298 (34.1%) had received postgraduate training in evidence-based medicine or clinical epidemiology. 41.8% of participants rated their confidence in their ability to interpret diagnostic data as in the middle (“3”) of the 5-point scale.

**Table 2:** Characteristics of participants.

		Text-only	Nomogram	Probability modifying plot	Natural frequencies
		N = 255	N = 194	N = 218	N = 207
Age group	20–29	30 (11.8%)	39 (20.1%)	32 (14.7%)	46 (22.2%)
	30–34	52 (20.4%)	49 (25.3%)	39 (17.9%)	37 (17.9%)
	35–39	49 (19.2%)	36 (18.6%)	46 (21.1%)	42 (20.3%)
	40–44	51 (20.0%)	21 (10.8%)	30 (13.8%)	26 (12.6%)
	45–49	27 (10.6%)	21 (10.8%)	21 (9.6%)	13 (6.3%)
	50–54	23 (9.0%)	10 (5.2%)	22 (10.1%)	18 (8.7%)
	55–59	16 (6.3%)	11 (5.7%)	10 (4.6%)	12 (5.8%)
	60+	7 (2.8%)	7 (3.6%)	18 (8.3%)	13 (6.3%)
Sex	Male	153 (60.0%)	112 (57.7%)	122 (56.0%)	124 (59.9%)
Professional status	GP	30 (11.9%)	23 (11.9%)	29 (13.4%)	11 (5.4%)
	Consultant	89 (35.2%)	57 (29.5%)	69 (31.8%)	62 (30.2%)
	Trainee GP	14 (5.5%)	4 (2.1%)	6 (2.8%)	11 (5.4%)
	Trainee junior doctor	91 (36.0%)	88 (45.6%)	85 (39.2%)	88 (42.9%)
	Other	29 (11.5%)	21 (10.9%)	28 (12.9%)	33 (16.1%)
Postgraduate training in evidence-based medicine of clinical epidemiology	Yes	89 (35.3%)	66 (34.9%)	79 (36.6%)	64 (31.5%)
	No	163 (64.7%)	123 (65.1%)	137 (63.4%)	139 (68.5%)
How confident are you in your ability to interpret data (e.g. sensitivity, specificity) from diagnostic research studies on the performance of diagnostic tests?	1 (not at all)	22 (8.7%)	18 (9.5%)	17 (7.8%)	13 (6.4%)
	2	66 (26.2%)	54 (28.4%)	51 (23.5%)	54 (26.6%)
	3	98 (38.9%)	76 (40.0%)	94 (43.3%)	97 (47.8%)
	4	63 (25.0%)	37 (19.5%)	52 (24.0%)	35 (17.2%)
	5 (extremely)	3 (1.2%)	5 (2.6%)	3 (1.4%)	4 (2.0%)
Mean (SD) number of correct answers to questions about diagnostic tests^‡^		2.1 (1.3)	2.3 (1.3)	2.2 (1.2)	2.2 (1.3)

^‡^ See **Table A in S2 File** for more detailed breakdown.

### Primary outcome

Participants who were allocated to the probability modifying plot and natural frequency tree arms were more likely to say that they would treat the patient (ORs 1.49, 95% CI 1.02, 2.16 and 1.43, 95% CI 0.98, 2.08 respectively) compared to the text only arm (**Table 3**). The mean post-test probability given by those who said that they would treat was 81.4% (95% CI 80.3%, 82.5%), compared with 54.4% (95% CI 49.8%, 59.1%) among those who said “Do not treat” and 71.4% (69.2%, 73.7%) among those who would have requested a biopsy (p<0.001). Adjusting for the participants’ estimated post-test probabilities attenuated the effects of the probability modifying plot and natural frequency tree on expressed intention to treat the patient (adjusted odds ratios 0.99, 95% CI 0.65, 1.50 and 1.28, 95% CI 0.84, 1.94, respectively), suggesting that the effects of these presentation on intended treatment were via their effects on doctors’ ability to calculate the post-test probability of disease correctly.

**Table 3:** Results by randomisation to method of presenting diagnostic test results.

	Clinical management					Post-test probability			
	Treatment	Biopsy	No treatment	Don’t know or blank	Odds Ratio (95% CI)	Correct	Incorrect	Don’t know or blank	Odds Ratio (95% CI)
Text-only (N = 255)	142 (55.7%)	79 (31.0%)	31 (12.2%)	3 (1.2%)	1.00 (reference)	30 (11.8%)	218 (85.5%)	7 (2.8%)	1.00 (reference)
Nomogram (N = 194)	112 (57.7%)	56 (28.9%)	24 (12.4%)	2 (1.0%)	1.09 (0.75, 1.58)	50 (25.8%)	137 (70.6%)	7 (3.6%)	2.60 (1.58, 4.29)
Probability modifying plot (N = 218)	142 (65.1%)	57 (26.2%)	16 (7.3%)	3 (1.4%)	1.49 (1.02, 2.16)	87 (39.9%)	129 (59.2%)	2 (0.9%)	4.98 (3.12, 7.95)
Natural frequencies (N = 207)	133 (64.3%)	55 (26.6%)	18 (8.7%)	1 (0.5%)	1.43 (0.98, 2.08)	73 (35.3%)	129 (62.3%)	5 (2.4%)	4.09 (2.54, 6.58)

A correct response to the vignette-based question about post-test probability was given by 240/874 (27.4%) participants. There was clear evidence (p<0.001) that the proportion of participants answering correctly differed between trial arms. Compared with the text-only arm, participants allocated to the probability modifying plot arm were most likely to give the correct answer (OR 4.98, 95% CI 3.12, 7.95) followed by the natural frequency arm (4.09, 95% CI 2.54, 6.58) and the nomogram arm (2.60, 95% CI 1.58, 4.29) (**Table 3**). For the natural frequency arm, a substantial proportion of participants (173/206, 84.0%) correctly identified that 300 patients with Green syndrome had a positive test result. Odds ratios were similar after adjustment for participant characteristics (age, sex, professional status, confidence level and postgraduate training): modifying plot arm, OR = 5.23 (95% CI 3.24, 8.45); natural frequency arm, OR = 4.23 (95% CI 2.59, 6.90); nomogram arm, OR = 2.68 (95% CI 1.61, 4.45). A sensitivity analysis allowing a wider margin of error (+/-3%) found very similar results (odds ratios 2.68, 95% CI 1.67, 4.29, 5.23, 95% CI 3.35, 8.16, 3.88, 95% CI 2.46, 6.11 for the nomogram, probability modifying plot and natural frequency tree arms respectively).

### Baseline knowledge

There was little variation in the mean number of correct answers to questions about baseline knowledge across the trial arms, and similar proportions of participants in each arm gave correct answers to all these questions (see **Table 1** and **Table A in S2 File** for more detailed breakdown of performance on questions by arm allocation) indicating that the randomization method was adequate. In univariable analyses (**Table B in S2 File**), the total number of questions answered correctly was associated with age (lower score in the oldest age group), postgraduate training (higher score for those who answered ‘yes’), and self-rated degree of confidence (lowest score for those who were least confident). When all of the factors shown in **Table B in S2 File** were included in a multivariable model, self-rated degree of confidence was the only factor associated with the total number of questions answered correctly (p<0.001). If this factor was excluded, professional status (p = 0.03) and postgraduate training (p = 0.05) were independently associated with the total number of questions answered correctly.

### Optional further tutorials

A similar proportion of participants in each arm (36.2% overall) proceeded to the optional tutorial (**Fig 1**). Participants who scored more highly in the five questions about baseline knowledge were more likely to proceed to the tutorial (OR 1.18, 95% CI 1.06, 1.31 per correct answer), as were participants who were more confident in their ability to interpret data from diagnostic research studies (1.22, 95% CI 1.05, 1.42 per response category). Hospital consultants (0.61, 95% CI 0.38, 0.99) and trainees (0.61, 95% CI 0.39, 0.97) were less likely to take the optional tutorial than GPs. Results of post-tutorial test by choice of tutorial are shown in **Table C in S2 File.** No differences were observed in the change score by type of tutorial format. There was little evidence of associations with age, sex or postgraduate training. The magnitude of these estimated associations was little changed when all the factors mentioned above were included in a multivariable model.

## Discussion

This large randomised controlled trial showed that doctors randomised to diagnostic test information presented using natural frequencies or a probability modifying plot were more likely to decide to actively treat a patient and calculate the correct post-test probability of disease than those randomised to information presented as test characteristics or using a Fagan’s nomogram. The effects of these presentations on doctors’ intention to treat actively (rather than order a more invasive biopsy procedure or not treat) appeared mediated by their effects on doctors’ ability to calculate the post-test probability of disease. We believe that this is the first study to demonstrate such an effect on reported clinical behaviour. If the responses to this hypothetical example also apply to real-world clinical decisions, then these results suggest that presentation of clinical diagnostic data could be easily improved with the use of computer assisted decision support algorithms that present test characteristics in more accessible ways.

We have shown, like other previous studies, that doctors are generally poor at using the results of a diagnostic test in relation to pre-existing information on a patient to calculate the post-test probability of disease. Younger age, hospital doctor status, and postgraduate education were all associated with better baseline knowledge. Self-rated confidence was positively associated with better baseline knowledge but doctors who scored worse on the knowledge quiz were less likely to undertake the optional tutorial, thereby contributing to the “inverse knowledge” gap.

We have undertaken a systematic review of the interpretation of diagnostic test studies by health professionals and identified six previous randomised controlled trials, of which two tested doctors’ ability to use Bayesian reasoning and four compared other presentation formats (see **Appendix 2**) [7,12–16]. They generally tested knowledge or the ability to correctly predict the post-test probability either comparing different measures of accuracy or different presentation formats (probability versus natural frequency). Providing further information was better than not doing so, with natural frequencies out-performing simple percentages. One study showed that a visual aid was better regardless of what data were presented [14]. Only one study [16] also went on to test how this information may change clinical behaviour. It did not find that the presentation of data on sensitivity and specificity, with or without definitions, altered reported clinical management but participants with a lower post-test probability for pertussis infection in a 5 month old girl were more likely to stop erythromycin therapy and discharge the patient form hospital. In this study we found that the probability modifying plot slightly out-performed the natural frequencies both in terms of the correct estimation of the post-test probability and importantly influencing clinical behaviour.

### Strengths & weaknesses

The relatively large sample size allowed us to compare four different intervention arms. We used a hypothetical disease example because we wished to avoid any influence of pre-existing attitudes or knowledge towards existing tests and clinical conditions. We chose a scenario where failure to treat a true case could result in long-term serious disability but alternative diagnostic measures required an invasive biopsy with obvious morbidity and potential mortality. Because the scenario was artificial, participants may not have applied the same reasoning as they would for a real clinical condition. However, as there was no “correct answer” to our management question we have no reason to believe that the doctors completing the module were biased or altered their treatment threshold compared to a real world clinical problem. It is possible that participants did not take the scenario seriously. This should have biased our results to the null. A potential limitation of our study is that participants were self-selected and most likely represent doctors most interested in continuing education. However the finding that particular ways to present information on text accuracy are more helpful to doctors is likely to be generalizable to less knowledgeable or motivated groups. We only tested the different types of presentation at a fixed pre-test probability of 50%. We cannot therefore be certain that our findings would generalize to lower or higher pre-test probabilities though in these scenarios diagnostic information is less likely to alter a clinical diagnosis or treatment decisions.

Whilst doctors vary in their individual trade-offs between treatment benefits and harms, greater diagnostic accuracy is likely to improve the quality of their treatment decisions. With the advance of sophisticated clinical support systems [20], future randomised controlled trials should compare real care decisions based on the provision of additional diagnostic data to routine test ordering behaviour. Medical schools and the Royal Colleges should ensure that medical students and doctors are assessed in their ability to interpret diagnostic data. Such skills are not merely of academic interest but also influence patient care and health care costs.

## Supporting Information

S1 File Appendix 1: Second- post-tutorial test; Appendix 2: Summary of six previous RCTs that have tested the interpretation of diagnostic test data. (DOCX)

S2 File Table A: Evaluation of baseline knowledge of diagnostic concepts, by trial arm; Table B: Odds ratios for answering 3+ *versus* 0–2 questions correctly, by participant characteristics; Table C: Results of post-tutorial test, by choice of tutorial. (DOCX)
