# Supplementary material for: Presentation of Diagnostic Information to Doctors May Change Their Interpretation and Clinical Management: A Web-Based Randomised Controlled Trial
Source: PLoS One. 2015 Jul 6;10(7):e0128637. doi: 10.1371/journal.pone.0128637 (PMC4492926; doi:10.1371/journal.pone.0128637)
Supplement: S1 File — (DOCX) [file pone.0128637.s001.docx]

**Appendix 1: Second- post-tutorial test**

The following are performance values for a new diagnostic test: sensitivity 50%; specificity 98%; positive predictive value 86%; negative predictive value 89%. All participants were asked the following 5 questions:

1) What percentage of patients with the disease are correctly identified by the test?

2) Given a positive test result, what is the risk of having the disease?

3) What is the false positive rate of this test?

4) What percentage of patients with a negative test will still have the disease?

5) Is this test more useful for ruling out or ruling in the diagnosis of the disease?

Answers to questions 1) – 4) could be entered into a text box beneath each question, which participants were instructed to leave blank for “don’t know”. Three possible answers to question 5) were presented as mutually-exclusive tick-boxes: “Ruling out”; “Ruling in”; and “Don’t know”.

On the final web page, all participants were presented with answers to the 5 questions as follows: 1) 50%; 2) 86%; 3) 2%; 4) 11%; 5) “Ruling in”.

**Appendix 2: Summary of six previous RCTs that have tested the interpretation of diagnostic test data**

| Author | Year | Population | Design | results |
| --- | --- | --- | --- | --- |
| Steurer | 2002 | 263 general practitioners in Switzerland | Test result only, result plus sensitivity and specificity, result plus likelihood ratio (described in words) | Test results alone resulted in over-interpretation and this was more moderate if additional information was provided |
| Puhan | 2005 | 183 physicians attending medical education conference in Switzerland | Group 1: Sensitivity and specificity  Group 2: Positive or negative likelihood ratio defined in words  Group 3: simple graphic of 5 circles based on LR. | No real difference regardless of presentation format but in two of the vignettes the graphical format was better |
| Bramwell | 2006 | 43 pregnant women, 42 midwives, 41 Obstetricians | Information on sensitivity and 1-specificity (as FPR) reported in words (terms not used) or as natural frequencies | Presentation with natural frequencies was better than with percentages but only for the obstetricians  Probability format (sensitivity and FPR as words):  - None of the midwives and 1 (5%) of the obstetricians gave the correct answer.  - 46% of midwives and 76% of obstetricians overestimated the PPV  - 55% of midwives and 19% of obstetricians underestimated the PPV.  Natural frequency format:  - None of the midwives and 13 (65%) of the obstetricians gave the correct answer.  -35% of midwives and 15% of obstetricians overestimated the PPV  -65% of midwives and 20% of obstetricians underestimated the PPV |
| Sox | 2009 | 653 US Pediatricians | Group 1: no information  Group 2: sensitivity and specificity  Group 3: sensitivity and specificity with definitions (normalized frequencies) | There was no difference (p=0.16) in the mean post-test probability between groups 1 and 2 (38% and 41%). Group 3 (45%) had a significantly higher mean post-test probability than group 1 (p=0.007). |
| Agoritsas | 2011 | 1361 physicians in Geneva, Switzerland | Sensitivity and specificity described in words and numerical frequencies (terms not used) for very accurate test (sensitivity and specificity 99%)  Doctors randomised to receive information on different prevalence (1%, 2%, 10%, 25%, 95%) and no information | Most respondents (66.7% to 80.3%)  selected a post-test probability of  95–99.9%, regardless of the prevalence of disease and even when no information on prevalence was provided.  Estimated that 9.1% (95% CI 6.0–14.0) of respondents knew how to assess correctly the post-test probability. This proportion did not vary with clinical experience or practice setting. |
| Garcia-Retamero | 2013 | 81 doctors and 81 patients from Granada, Spain | Factorial design: Information on sensitivity FPR and prevalence reported in words (terms not used) or as natural frequencies. Half participants received this information depicted with visual aids | Natural frequencies were superior to probabilities but visual aid resulted in best results (post-test probability proportion correct):  Probabilities alone: 23%  Natural frequencies alone: 48%  Probabilities with visual aid: 68%  Natural frequencies with visual aid:73% |
