# Supplementary material for: Presentation of Diagnostic Information to Doctors May Change Their Interpretation and Clinical Management: A Web-Based Randomised Controlled Trial
Source: PLoS One. 2015 Jul 6;10(7):e0128637. doi: 10.1371/journal.pone.0128637 (PMC4492926; doi:10.1371/journal.pone.0128637)
Supplement: S2 File — (DOCX) [file pone.0128637.s002.docx]

**Table A: Evaluation of baseline knowledge of diagnostic concepts, by trial arm**

|  | Text only | Nomogram | Probability modifying plot | Natural frequencies |
| --- | --- | --- | --- | --- |
|  | N=255 | N=194 | N=218 | N=207 |
| Q1. What percentage of patients with the disease would be misdiagnosed? | | | | |
| Correct | 94 (36.9%) | 81 (41.8%) | 80 (36.7%) | 75 (36.2%) |
| Incorrect | 90 (35.3%) | 60 (30.9%) | 80 (36.7%) | 68 (32.9%) |
| Blank/don’t know | 71 (27.8%) | 53 (27.3%) | 58 (26.6%) | 64 (30.9%) |
| Q2. Given a positive test result, what is the probability of having the disease? | | | | |
| Correct | 107 (42.0%) | 104 (53.6%) | 108 (50.05) | 90 (43.5%) |
| Incorrect | 78 (30.6%) | 47 (24.2%) | 61 (28.0%) | 59 (28.5%) |
| Blank/don’t know | 70 (27.5%) | 43 (22.2%) | 48 (22.0%) | 58 (28.0%) |
| Q3. What is the false positive rate of this test? | | | | |
| Correct | 91 (35.7%) | 64 (33.0%) | 74 (33.9%) | 69 (33.3%) |
| Incorrect | 75 (29.4%) | 64 (33.0%) | 75 (34.4%) | 74 (35.8%) |
| Blank/don’t know | 89 (34.9%) | 66 (34.0%) | 69 (31.7%) | 64 (30.9%) |
| Q4. What percentage of patients with a negative test will still have the disease? | | | | |
| Correct | 102 (40.0%) | 93 (47.9%) | 96 (44.0%) | 100 (48.3%) |
| Incorrect | 87 (34.1%) | 55 (28.4%) | 71 (32.6%) | 50 (24.2%) |
| Blank/don’t know | 66 (25.9%) | 46 (23.7%) | 51 (23.4%) | 57 (27.5%) |
| Q5. Is this test more useful for ruling out or ruling in the diagnosis of the disease? | | | | |
| “Ruling out” (correct) | 100 (41.0%) | 80 (42.3%) | 91 (42.7%) | 81 (39.9%) |
| “Ruling in” | 70 (28.7%) | 56 (29.6%) | 44 (20.7%) | 62 (30.5%) |
| “Don’t know” | 74 (30.3%) | 53 (28.0%) | 78 (36.6%) | 60 (29.6%) |
| Total number of correct answers to Q1 – Q5 | | | | |
| 0 | 28 (11.0%) | 16 (8.3%) | 11 (5.1%) | 10 (4.8%) |
| 1 | 71 (27.8%) | 44 (22.7%) | 62 (28.4%) | 66 (31.9%) |
| 2 | 57 (22.4%) | 42 (21.7%) | 60 (27.5%) | 48 (23.2%) |
| 3 | 49 (19.2%) | 53 (27.3%) | 46 (21.1%) | 43 (20.8%) |
| 4 | 44 (17.3%) | 31 (16.0%) | 34 (15.6%) | 35 (16.9%) |
| 5 | 6 (2.4%) | 8 (4.1%) | 5 (2.3%) | 5 (2.4%) |

**Table B: Odds ratios for answering 3+ *versus* 0-2 questions correctly, by participant characteristics**

|  |  | 3+ correct/  total (%) | Odds ratio (95% CI) |
| --- | --- | --- | --- |
| Age group | 20-29 | 85/147 (42.2%) | 1.00 |
|  | 30-34 | 74/177 (41.8%) | 0.98 (0.63, 1.53) |
|  | 35-39 | 80/173 (46.2%) | 1.18 (0.76, 1.84) |
|  | 40-44 | 56/128 (43.8%) | 1.07 (0.66, 1.72) |
|  | 45-49 | 29/82 (35.4%) | 0.75 (0.43, 1.31) |
|  | 50-54 | 28/73 (38.4%) | 0.85 (0.48, 1.51) |
|  | 55-59 | 21/49 (42.9%) | 1.03 (0.53, 1.98) |
|  | 60+ | 9/45 (20.0%) | 0.34 (0.53, 1.01) |
| p value for trend |  |  | P=0.04 |
| Gender | Female | 144/363 (39.7%) | 1.00 |
|  | Male | 215/511 (42.1%) | 1.10 (0.84, 1.45) |
| Professional status | GP | 35/93 (37.6%) | 1.00 |
|  | Consultant | 123/277 (44.4%) | 1.32 (0.82, 2.14) |
|  | Trainee | 169/387 (43.7%) | 1.28 (0.81, 2.05) |
|  | Other | 32/117 (27.4%) | 0.62 (0.35, 1.12) |
| Postgraduate training in evidence-based medicine of clinical epidemiology. | No | 218/562 (38.8%) | 1.00 |
|  | Yes | 136/298 (45.6%) | 1.32 (1.00, 1.76) |
| How confident are you in your ability to interpret data (e.g. sensitivity, specificity) from diagnostic research studies on the performance of diagnostic tests? | 1 (not at all) | 15/70 (21.4%) | 1.00 |
|  | 2 | 77/225 (34.2%) | 1.91 (1.01, 3.60) |
|  | 3 | 156/365 (42.7%) | 2.74 (1.49, 5.02) |
|  | 4 | 101/187 (54.0%) | 4.31 (2.27, 8.16) |
|  | 5 (extremely) | 8/15 (53.3%) | 4.19 (1.31, 13.4) |
| p value for trend |  |  | P<0.001 |
| Proceeded to optional tutorial | No | 208/558 (37.3%) | 1.00 |
|  | Yes | 151/316 (47.8%) | 1.54 (1.16, 2.04) |

**Table C: Results of post-tutorial test, by choice of tutorial**

|  | | Slides only | Short video | Long video |
| --- | --- | --- | --- | --- |
| Q1. What percentage of patients with the disease are correctly identified by the test? | | | | |
| Correct | 93 (50.5%) | | 48 (59.3%) | 36 (70.6%) |
| Incorrect | 39 (21.2%) | | 22 (27.2%) | 8 (15.7%) |
| Blank/don’t know | 52 (28.3%) | | 11 (13.6%) | 7 (13.7%) |
| Odds Ratio (95% CI)^a^ | 1.00 | | 1.42 (0.84, 2.42) | 2.35 (1.20, 4.58) |
| Q2. Given a positive test result, what is the probability of having the disease? | | | | |
| Correct | 104 (56.5%) | | 47 (58.0%) | 37 (72.6%) |
| Incorrect | 25 (13.6%) | | 19 (23.5%) | 6 (11.8%) |
| Blank/don’t know | 55 (29.9%) | | 15 (18.5%) | 8 (15.7%) |
| Odds Ratio (95% CI)^a^ | 1.00 | | 1.06 (0.63, 1.80) | 2.03 (1.03, 4.02) |
| Q3. What is the false positive rate of this test? | | | | |
| Correct | 62 (33.7%) | | 31 (38.3%) | 21 (41.2%) |
| Incorrect | 57 (31.0%) | | 33 (40.7%) | 19 (37.3%) |
| Blank/don’t know | 65 (35.3%) | | 17 (21.0%) | 11 (21.6%) |
| Odds Ratio (95% CI)^a^ | 1.00 | | 1.22 (0.71, 2.10) | 1.38 (0.73, 2.60) |
| Q4. What percentage of patients with a negative test will still have the disease? | | | | |
| Correct | 84 (45.7%) | | 40 (49.4%) | 25 (49.0%) |
| Incorrect | 42 (22.8%) | | 25 (30.9%) | 19 (37.3%) |
| Blank/don’t know | 58 (31.5%) | | 16 (20.0%) | 7 (13.7%) |
| Odds Ratio (95% CI)^a^ | 1.00 | | 1.16 (0.69, 1.96) | 1.14 (0.62, 2.13) |
| Q5. Is this test more useful for ruling out or ruling in the diagnosis of the disease? | | | | |
| “Ruling in” (correct) | 104 (58.4%) | | 43 (53.8%) | 29 (59.2%) |
| “Ruling out” | 25 (14.0%) | | 22 (27.5%) | 14 (28.6%) |
| “Don’t know” | 49 (27.5%) | | 15 (18.8%) | 6 (12.2%) |
| Odds Ratio (95% CI)^a^ | 1.00 | | 0.83 (0.49, 1.41) | 1.03 (0.54, 1.96) |
| Correct answers to Q1 – Q5 | | | | |
| Yes | 27 (14.7%) | | 12 (14.8%) | 8 (15.7%) |
| No | 157 (85.3%) | | 69 (85.2%) | 43 (84.3%) |
| Odds Ratio (95% CI)^b^ | 1.00 | | 1.01 (0.48, 2.11) | 1.08 (0.46, 2.55) |
| Change in score (number of questions answered correctly after tutorial - number of questions answered correctly before tutorial) | | | | |
| Better | 68 (38.6%) | | 26 (33.3%) | 22 (46.8%) |
| No change | 37 (21.0%) | | 17 (21.8%) | 10 (21.3%) |
| Worse | 71 (40.3%) | | 35 (44.9%) | 15 (31.9%) |
| Odds Ratio (95% CI)^c^ | 1.00 | | 0.79 (0.45, 1.39) | 1.40 (0.73, 2.67) |

## ^a^ Odds of correct/odds of (incorrect + blank/don’t know)

## ^b^ Odds of yes/odds of no

## ^c^ Odds of better/odds of (no change + worse)
